# Simultaneous expansion microscopy imaging of proteins and mRNAs via dual-ExM

**DOI:** 10.1038/s41598-022-06903-3

**Published:** 2022-03-01

**Authors:** In Cho, Jae-Byum Chang

**Affiliations:** Department of Materials Science and Engineering, Korea Advanced Institute of Science and Technology (KAIST), Daejeon, 34141 Republic of Korea

**Keywords:** Microscopy, Confocal microscopy, Super-resolution microscopy

## Abstract

Simultaneous nanoscale imaging of mRNAs and proteins of the same specimen can provide better information on the translational regulation, molecular trafficking, and molecular interaction of both normal and diseased biological systems. Expansion microscopy (ExM) is an attractive option to achieve such imaging; however, simultaneous ExM imaging of proteins and mRNAs has not been demonstrated. Here, a technique for simultaneous ExM imaging of proteins and mRNAs in cultured cells and tissue slices, which we termed dual-expansion microscopy (dual-ExM), is demonstrated. First, we verified a protocol for the simultaneous labeling of proteins and mRNAs. Second, we combined the simultaneous labeling protocol with ExM to enable the simultaneous ExM imaging of proteins and mRNAs in cultured cells and mouse brain slices and quantitatively study the degree of signal retention after expansion. After expansion, both proteins and mRNAs can be visualized with a resolution beyond the diffraction limit of light in three dimensions. Dual-ExM is a versatile tool to study complex biological systems, such as the brain or tumor microenvironments, at a nanoscale resolution.

## Introduction

Simultaneous imaging of proteins and mRNAs in single specimen can provide more information than the imaging of only proteins or mRNAs^1^. For example, multiple studies have reported that mRNA levels are not always linearly proportional to protein expression levels^2–4^ due to translational regulation or the regulation of protein degradation^5^. The study of these regulations could be enabled by the simultaneous imaging of proteins and mRNAs in single specimen. Additionally, multiple studies have shown that a considerable fraction of mRNAs is localized in specific subcellular domains^6,7^. Such localization of mRNAs allows spatially restricted gene expression, which facilitates efficient local translations and rapid cellular responses to external stimuli^8^. Furthermore, the study of such mRNA localization could be enabled by the simultaneous imaging of mRNAs and cellular organelle proteins. Recently, multiple imaging techniques enabling the simultaneous visualization of both proteins and mRNAs have been developed^1,9,10^. However, these techniques have only been demonstrated in cultured cells or thin-tissue slices. Moreover, their imaging resolution is limited by the diffraction of light and is not sufficiently high to image nanoscale protein structures or to resolve two mRNA molecules located closer than the diffraction limit^11^.

Expansion microscopy (ExM) enables the super-resolution imaging of proteins or mRNAs in thick-tissue slices by physically expanding tissue slices and subsequently imaging them using conventional diffraction-limited microscopy^11–13^. ExM techniques can be divided into two groups—pre-gel labeling ExM and post-gel labeling ExM—depending on when labels are introduced to specimens. Post-gel labeling ExM techniques introduce labels after expansion, which achieve a deeper labeling depth and a smaller effective label size. However, the RNA signal integrity during the expansion process is hard to track, as the RNA can be lost or degraded during the expansion process. Additionally, the post-gel labeling ExM techniques require the modification of the labeling protocols, as the labeling is performed in charged polymer networks. However, pre-gel labeling ExM techniques perform regular labeling procedures and proceed to the ExM process. Pre-gel labeling ExM techniques can be an attractive option for the simultaneous imaging of both proteins and mRNAs for broader use, as their staining result can be easily examined after the staining and commercial protocol could be applied without any modification. However, the simultaneous imaging of proteins and mRNAs via pre-gel labeling expansion microscopy has not been demonstrated due to the lack of quantitative study on how much both immunofluorescence (IF) and fluorescence in-situ hybridization (FISH) signals would be retained after expansion.

Here, we demonstrate the simultaneous ExM imaging of both proteins and mRNAs in cultured cells and tissue slices through the optimization of a labeling procedure and a quantitative study on the degree of retention of mRNA FISH signals before and after expansion. In this study, first, we optimized labeling procedures to label both proteins and mRNAs with high fidelity. Second, we combined two ExM techniques, one developed for the ExM imaging of proteins and the other for the ExM imaging of mRNAs, to enable the simultaneous ExM imaging of both proteins and mRNAs. We show that the labeling and ExM process demonstrated here, termed dual-ExM, can visualize both the proteins and mRNAs from the same specimens at a lateral resolution beyond the diffraction limit of light.

## Results

### Process of dual-ExM and biomolecule labeling signal integrity

**Figure 1:**
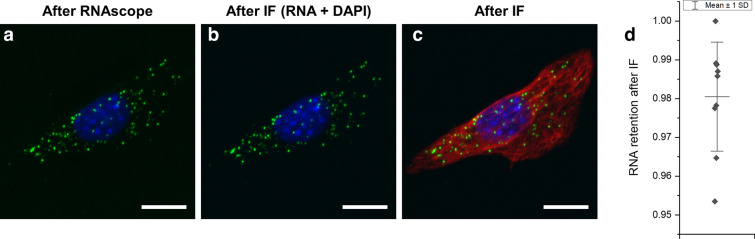
mRNA retention rate after immunostaining. (**a**) Maximum intensity projection (MIP) of a z-stack image of a NIH-3T3 cell, labeled with an RNAscope probe against *Polr2a* mRNA (green) and DAPI (blue). (**b**) Image of DAPI and *Polr2a* mRNA of the cell shown in (**a**) after immunostaining of vimentin. (**c**) Image of vimentin (red), *Polr2a* mRNA (green), and DAPI (blue) after immunostaining. (**d**) mRNA retention rate after IF. The numbers of mRNA puncta before and after IF were compared. Whisker range shows mean value ± standard deviation, n = 9 cells from three different cell cultures. Scale bars in (**a**–**c**): 20 μm.

For the labeling of both proteins and mRNAs, we first determined the order of the labeling procedures in cultured cells. We defined two procedures: first, mRNAs of cultured cells were labeled with FISH probes, and subsequently, proteins of the same cells were labeled with antibodies, a process we termed FISH-IF; second, proteins of cells were labeled with antibodies, and then mRNAs of the same cells were labeled with FISH probes, a process we termed IF-FISH. We first tested FISH-IF. For mRNA FISH, we tested two commercially available and extensively used mRNA labeling techniques, which were hybridization chain reaction (HCR) 3.0^14^ and RNAscope^15^. To study the effect of the FISH procedure on subsequent antibody labeling, we applied the HCR or RNAscope procedures to cultured cells without probes; subsequently, we labeled vimentin of the cells with antibodies. As shown in Supplementary Fig. 1, vimentin filaments of the cells processed with the HCR procedure were highly punctuated, possibly due to the denaturation or the aggregate formation of vimentin during the 24-h long formamide treatment included in the HCR procedure. Interestingly, vimentin filaments were more continuous in the cells processed with the RNAscope protocol, which included 4-h long formamide treatment. Subsequently, we studied whether the process of immunolabeling of proteins affects the FISH probes bound to their target mRNA molecules when immunolabeling is performed after FISH. More than 98% of *Polr2a* mRNA puncta were retained after the immunostaining of proteins (Fig. 1).

**Figure 2:**
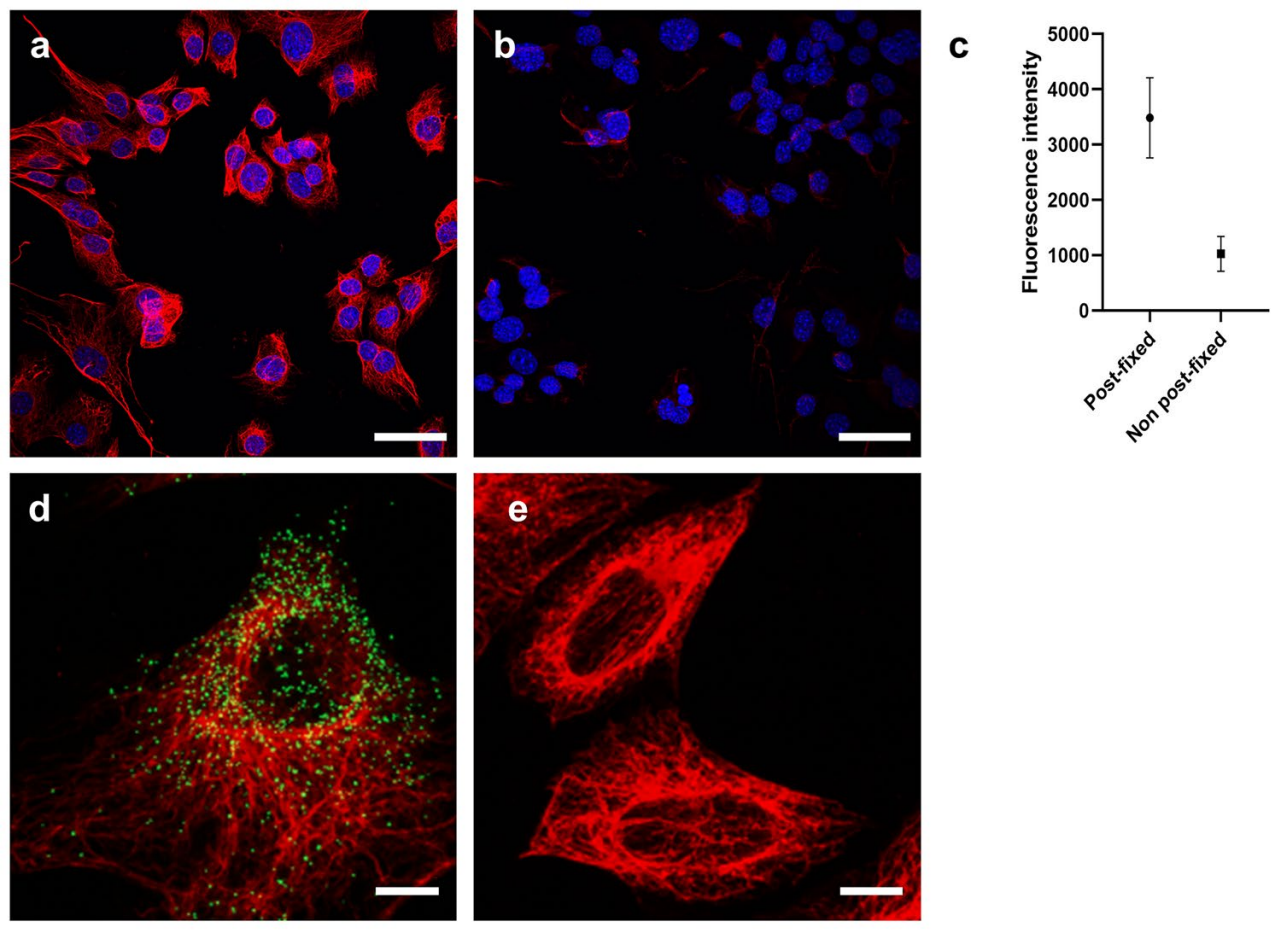
Effect of a post-fixation step between IF and FISH in the IF-FISH process. (**a**,**b**) Maximum intensity projection (MIP) of a z-stack image of NIH-3T3 cells labeled with an antibody against vimentin (red) and DAPI (blue). After antibody labeling, cells were either post-fixed or not post-fixed and then proceeded to FISH. (**a**) Cells post-fixed after IF. Vimentin signals are visible. (**b**) Cells not post-fixed after IF. Most of the vimentin signals were lost. (**c**) Relative fluorescence intensity of vimentin fibers of the cells shown in (**a**) and (**b**). (**d**,**e**) Validation of the mRNA labeling of post-fixed cells. (**d**) MIP image of a NIH-3T3 cell, labeled with an RNAscope probe against *Gapdh* mRNA (green) and antibody against vimentin (red). (**e**) MIP image of NIH-3T3 cells, labeled with an RNAscope negative control probe (*DapB*) and antibody against vimentin. Scale bars in (**a**,**b**) 50 μm; (**d**,**e**) 10 μm.

Next, we tested the IF-FISH process; we tested whether the IF-FISH process visualizes both proteins and mRNAs. We found that specimens needed to be chemically fixed after IF and before FISH. Without such post-fixation, IF signals were lost after the FISH process, possibly due to the detachment of antibodies from their antigens or the digestion of antibodies during the FISH process (Fig. 2a–c). Furthermore, we confirmed that the additional fixation step after immunostaining, but before the FISH process, does not result in the non-specific binding of FISH probes (Fig. 2d–e). As such, IF-FISH and FISH-IF both successfully visualized both proteins and mRNAs; however, both techniques had distinct advantages and disadvantages when compared to each other. Multiple reports have shown that the permeabilization of cells, which is required in the immunolabeling process, induces the loss of RNA molecules^16–20^, resulting in a reduced number of mRNA puncta in the IF-FISH process. However, it has been reported that some antibodies do not bind to their targets when antibodies are introduced to specimens after the RNAscope labeling process^1^, resulting in the limited range of proteins to be imaged in the FISH-IF process. Between FISH-IF and IF-FISH, we used FISH-IF for the development of dual-ExM, as it better preserved both protein and mRNA signals.

**Figure 3:**
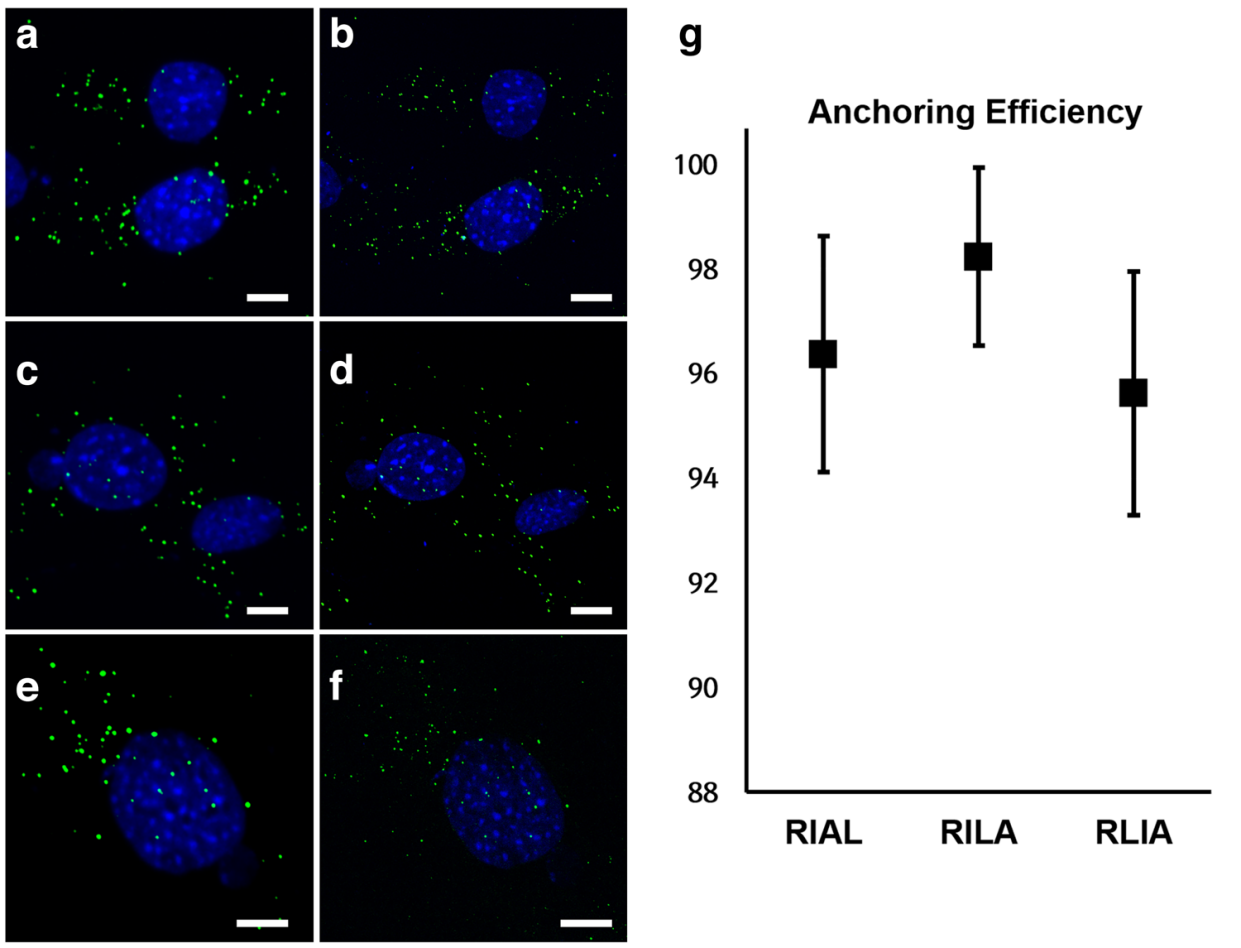
mRNA anchoring efficiency. (**a**) Maximum intensity projection (MIP) of a z-stack image of NIH-3T3 cells, labeled with RNAscope probes against *Ppib* mRNA (green) and DAPI (blue). (**b**) Same cells shown in (**a**) but after expansion via the RIAL process. (**c**) Different cells prepared in the same way as (**a**). (**d**) Same cells shown in **c** but after expansion via the RILA process. (**e**) Different cells prepared in the same way as (**a**) and (**c**). (**f**) Same cells shown in **e** but after expansion via the RLIA process. (**g**) mRNA anchoring efficiency after expansion prepared in three different processes. The numbers of *Ppib* mRNA puncta were compared before and after expansion. (black square, mean; lines, ± one standard deviation. n = 9 for each process). Scale bars in (**a**–**f**): 10 μm. All length scales are presented in pre-expansion dimensions.

Subsequently, we combined two ExM procedures, each of which was developed for imaging of either proteins or mRNAs. The ExM imaging of antibody-labeled proteins, in short, proExM, begins with the labeling of proteins with primary antibodies and fluorophore-conjugated secondary antibodies. Following staining, 6-((acryloyl)amino)hexanoic acid (acryloyl-X, SE; abbreviated AcX) is applied to the specimens to add a polymerizable acrylamide functional group to the amines on the proteins in the specimens^12^. Hence, the specimens were incubated in a gelation solution and incubated at 37 °C to form a swellable hydrogel inside the specimens. During the in situ gelation process, antibodies in the specimens are anchored to the hydrogel networks. The hydrogel-specimen composites are subsequently treated with a proteinase to digest all protein structures resisting expansion. After digestion, the hydrogel-specimen composites are expanded in deionized water^12^. The ExM imaging of FISH-labeled mRNAs, in short, ExFISH, is similar to proExM, except a different anchoring chemical is used^11^. Instead of AcX, which reacts with all amines, LabelX is used to make all nucleotides gel-anchorable. LabelX reacts with N7 of guanine of nucleic acids and adds a polymerizable acrylamide functional group^11^. To image both proteins and mRNAs after expansion, antibodies and FISH probes in specimens should be anchored to a hydrogel network. We first studied how the order of the application of the two anchoring chemicals, AcX and LabelX, affects the anchoring efficiency of FISH probes. Three procedures were tested with different orders of labeling and the treatment of anchoring chemicals: first, FISH labeling of mRNAs, immunostaining of proteins, AcX treatment, and subsequently LabelX was applied, a process we termed RIAL. Second, FISH labeling of mRNAs, immunostaining of proteins, LabelX treatment, and then AcX was applied, a process we termed RILA. Third, FISH labeling of mRNAs, LabelX treatment, immunolabeling of proteins, and AcX was applied, a process we termed RLIA. For these three procedures, the number of mRNA puncta right after the labeling of mRNAs with RNAscope probes and after expansion were compared (see “Materials and methods”). As shown in Fig. 3, the three procedures showed mRNA retention rates over 95%, indicating that the order of applications of LabelX and AcX did not significantly affect the efficiency of anchoring the FISH probes to a hydrogel network.

### Dual-ExM imaging of cultured cells

**Figure 4:**
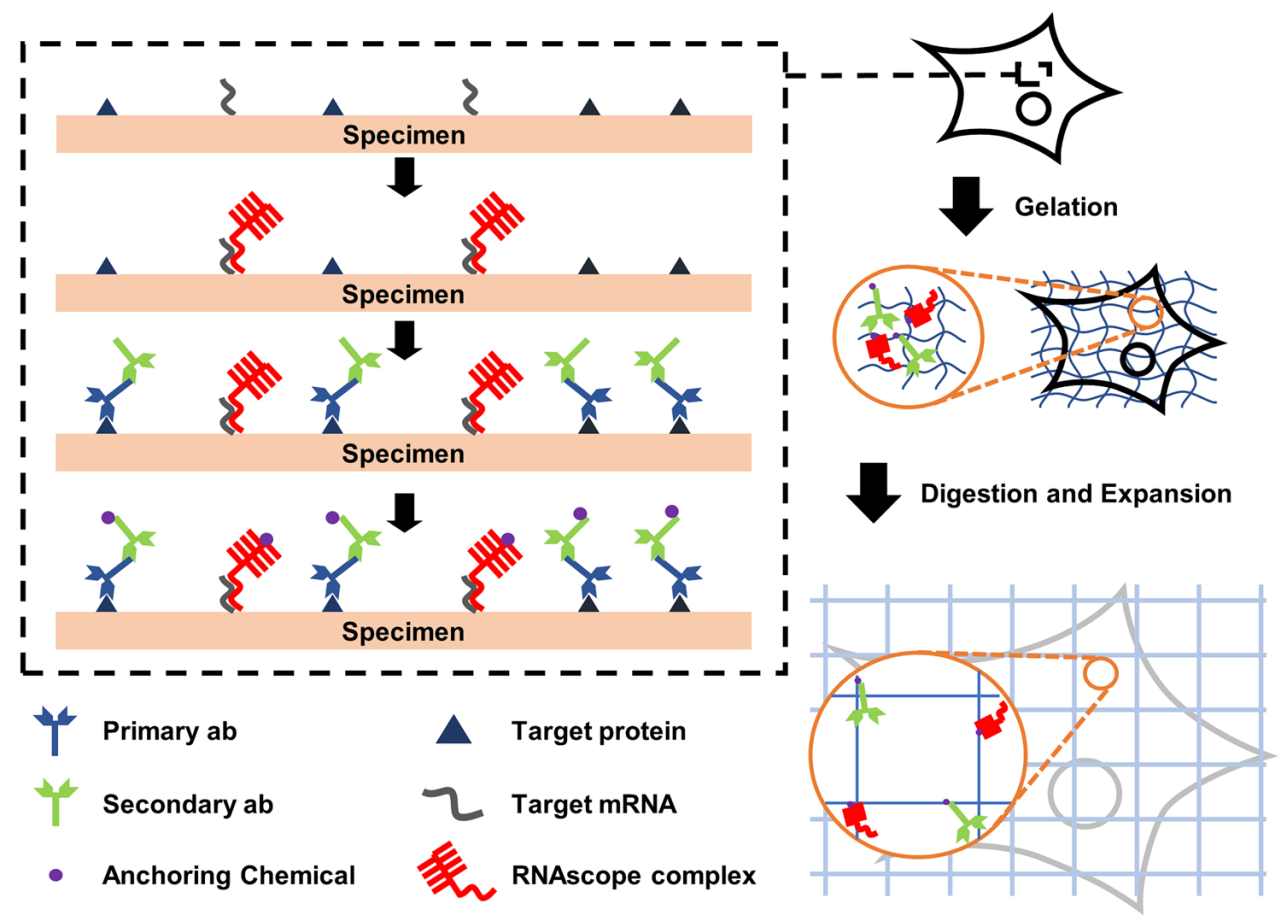
Workflow of dual ExM for the simultaneous super-resolution imaging of proteins and mRNAs. Chemically fixed specimens are first labeled with RNAscope FISH probes and antibodies. Then, anchoring chemicals, which are LabelX and AcX, are applied to the specimens. The specimens are embedded in a swellable hydrogel, digested, and expanded in deionized water.

**Figure 5:**
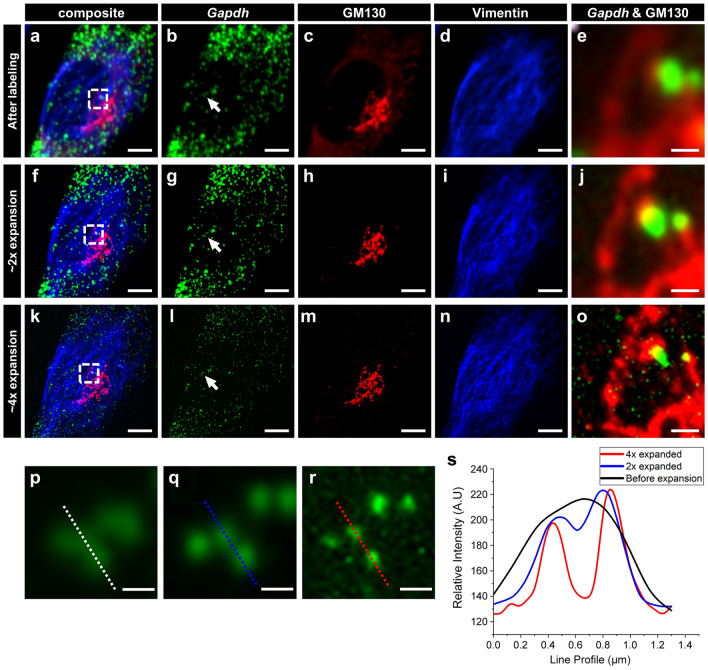
Dual ExM imaging of RNAs and proteins in cultured cells. (**a**–**o**) MIP of z-stack images of NIH-3T3 cells, labeled with an RNAscope probe against *Gapdh* mRNA (green) and antibodies against cis-golgi marker GM130 (red) and vimentin (blue). (**a**–**e**) Before expansion; (**f**–**j**) after 2.1-fold expansion; (**k**–**o**) after 3.7-fold expansion; (**a**,**f**,**k**) images showing all three channels; (**b**,**g**,**l**) images showing only *Gapdh* mRNA; (**c**,**h**,**m**) images showing only GM130; (**d**,**i**,**n**) images showing only vimentin; (**e**,**j**,**o**) magnified view of the boxed regions in (**a**,**f**,**k)**. (**p**–**r**) Magnified views of *Gapdh* mRNA marked by the white arrows in (**b**,**g**,**l**). (**s**) Line intensity profiles of *Gapdh* mRNA puncta shown in (**p**,**q**,**r)**. Scale bars: (**a**–**d**), (**f**–**i**), (**k**–**n**) 5 μm; (**e**, **j**, **o)** 1 μm; (**p**–**r**) 0.5 μm. All length scales are presented in pre-expansion dimensions.

After verifying that over 95% of the RNA puncta were retained during the immunostaining and subsequent expansion process, we performed simultaneous ExM imaging of both proteins and mRNAs in cultured NIH-3T3 cells, as described in Fig. 4. *Gapdh* mRNA and two proteins, including intermediate filament vimentin and Golgi protein GM130, were labeled with an RNAscope probe against *Gapdh* mRNA and antibodies. Labeled cells were then expanded 2.1-fold and 3.7-fold in 1 × PBS and 0.1 × PBS, respectively. The expansion yielded a dramatic improvement in the resolution of both proteins and mRNAs (Fig. 5). When the mRNA puncta, shown in Fig. 5b,g,l, were compared, it was clear that the size of the mRNA puncta decreased. Consequently, closely positioned mRNA puncta that were unresolved before expansion became resolved after expansion (Fig. 5p–s). Such a change in the size of mRNA puncta is due to the decrease in the effective size of the point-spread function (PSF) of microscopy. The structures on microscopy images are the result of convolution between physical structures in the specimens and the PSF of microscopy. In this study, FISH probes were applied before gel synthesis; FISH probes anchored in the hydrogel were also expanded with the hydrogel expansion. If these expanded FISH probes were imaged with the same microscopy used for the pre-expansion imaging and the resulting images were resized to the pre-expansion length scale, the size of the mRNA puncta in the post-expansion images should be smaller than that in the pre-expansion images due to the decrease in the effective PSF. A Golgi apparatus visualized by the staining of GM130 was also better resolved after the expansion (Fig. 5c,h,m). Similarly, single vimentin fibers were resolved after the fourfold expansion (Fig. 5d,i,n). Simultaneous dual imaging of mRNAs and proteins showed better information about where mRNA molecules are localized (Fig. 5e,j,o). In addition to imaging single mRNA target with multiple proteins, we further validated that the whole process is also compatible with multiple mRNA targets with immunofluorescence (Supplementary Fig. 2).

### Dual-ExM imaging of mouse brain tissue

**Figure 6:**
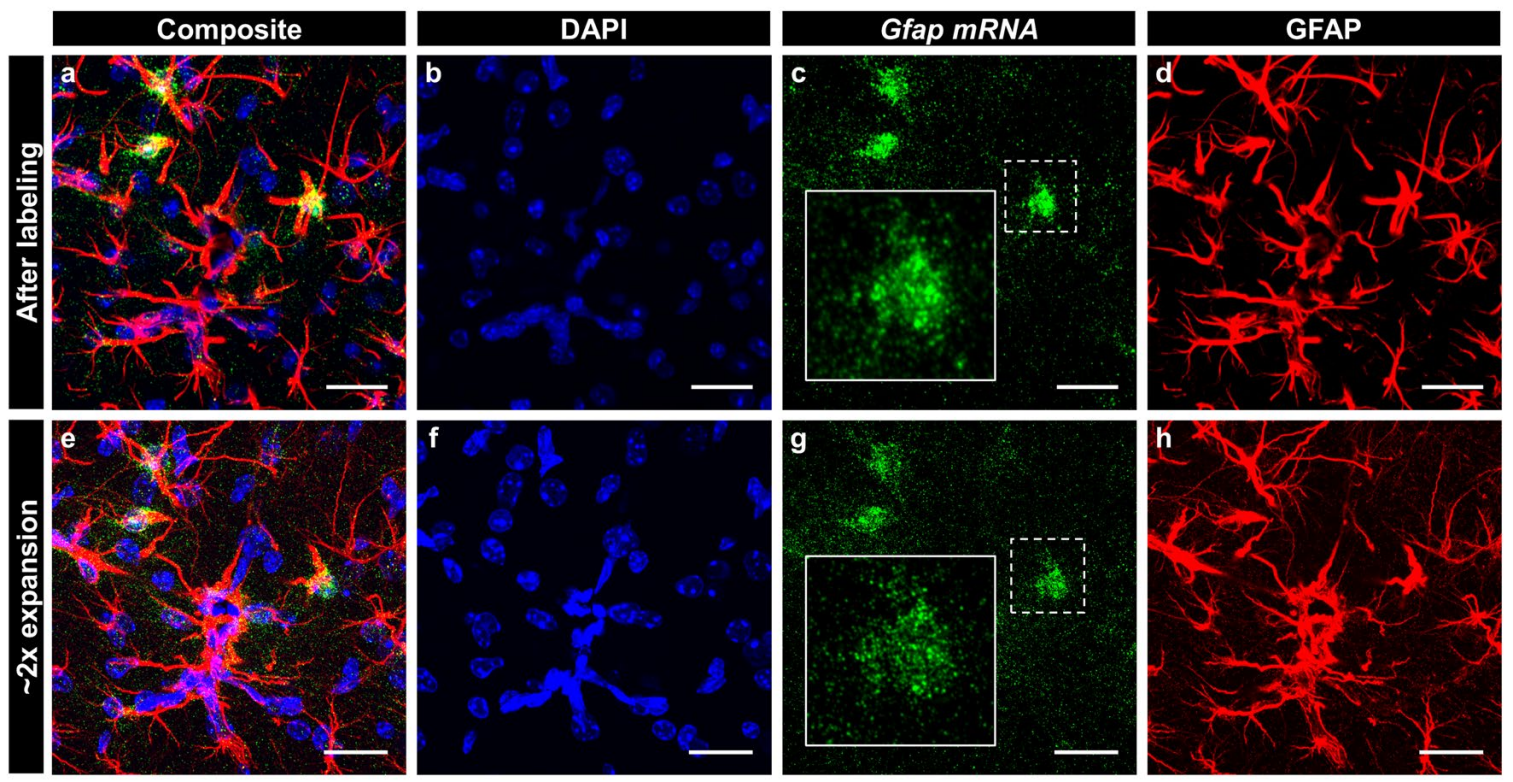
Dual ExM images of mRNAs and proteins in a fixed mouse brain slice. (**a**–**h**) MIP of z-stacks of a mouse brain slice, labeled with an RNAscope probe against *Gfap* mRNA (green), antibody against GFAP (red) and DAPI (blue). (**a**–**d**), before expansion; (**e**–**h**), after 2.1-fold expansion; (**a**,**e**) images showing all three channels, containing DAPI, *Gfap* mRNA, and GFAP protein. (**b**–**d**,**f**–**h**) images showing only one channel. (**b**,**f**) DAPI; (**c**,**g**) *Gfap* mRNA; (**d**,**h**) GFAP. White boxes in (**c**,**g**) show magnified views of the respective white dotted boxes. Scale bars in (**a**–**h**): 25 μm. All length scales are presented in pre-expansion dimensions.

We subsequently applied the super-resolution simultaneous imaging of proteins and mRNAs to thick brain slices. We first confirmed that RNAscope could visualize mRNA puncta inside thick brain slices, as the RNAscope protocol is designed for the mRNA imaging of thin tissue slices. However, with minor changes, such as elongated digestion and washing (see “Materials and methods” for details), mRNA puncta located at a depth of 50 μm from the surface of a mouse brain slice were successfully visualized (Supplementary Fig. 3). We labeled *Gfap* mRNA and proteins of a 200-μm-thick brain slice with the modified RNAscope protocol, followed by immunostaining of GFAP. As shown in Fig. 6a–d, both the GFAP proteins and mRNAs were clearly labeled. Interestingly, the expression level of *Gfap* mRNA was highly heterogeneous between cells, as indicated by the variation in the number of mRNA puncta in each GFAP-positive cell^21^. Again, with the modified RNAscope labeling protocol, *Gfap* mRNA puncta were clearly visualized over the 200-μm-thick brain slice. Hence, we performed the ExM procedure on the brain slice and expanded the brain slice 2.1-fold in 1 × PBS (Fig. 6e–h). As observed with the cultured cells shown in Fig. 5, individual *Gfap* mRNA puncta were more clearly resolved after the expansion, enabling higher-precision counting of the number of mRNAs. Due to the clearing of the tissue during the ExM process, we successfully demonstrated a three-dimensional (3D) visualization of both GFAP protein and *Gfap* mRNA (Supplementary Video 1).

We mainly demonstrated dual-ExM based on FISH-IF; however, some protein targets that are not properly labeled through the FISH-IF process can be labeled with the IF-FISH process and expanded with the same ExM procedure. As shown in Supplementary Fig. 4 and Supplementary Fig. 5, IF-FISH, combined with ExM, would also successfully visualize both proteins and mRNAs at an improved resolution; even if a fraction of mRNA signals were lost during the IF process.

## Discussion

In this study, we demonstrated the simultaneous super-resolution imaging of both mRNAs and proteins using commercially available labeling techniques and chemicals. We found that FISH labeling of mRNAs followed by the IF labeling of proteins induces the successful visualization of both molecular species after expansion. We quantified the degree of mRNA retention and found that most mRNAs (> 95%) were retained after the IF labeling and expansion process. Furthermore, we showed that post-fixation was required when the IF labeling of proteins was followed by the RNA FISH labeling to prevent the loss of the IF signals after the RNA FISH process. The series of techniques demonstrated here, which we termed dual-ExM, has multiple advantages: First, it does not use any special chemical; instead, it uses only commercially available chemicals, making it easily accessible to conventional labs. Second, FISH probes were applied first, and subsequently IF labeling was performed; hence, the FISH labeling process was not affected by the subsequent IF labeling process. Lastly, conventional super-resolution microscopy techniques, such as single molecule localization microscopy (SMLM)^22–24^, provide resolution on the order of tens of nanometers, while the application of such techniques to acquire 3D images from thick opaque tissue slices is often challenging due to the scattering of lights inside opaque specimens. In such a case, the dual-ExM procedure could be a simple way of achieving resolution beyond the diffraction limit of light, as tissue slices become transparent during the expansion process. Therefore, the dual-ExM simultaneously enables the sub-diffraction limit 3D imaging of multiple biomolecules from opaque tissue slices, with the axial resolution improved by the expansion factor, as shown in Fig. 6 (projected z-stack images obtained at the surface to 60 μm from the surface, using the pre-expansion dimension), and Supplementary Video 1 (3D rendered from images obtained at 10–30 μm from the surface, using the pre-expansion dimension). Moreover, when combined with lattice light-sheet microscopy, the axial resolution can be further improved^25^; therfore, the mRNAs can be localized in a more precise axial context.

Dual-ExM could be improved further in some aspects. First, the fluorescent signal intensity, especially the FISH signal, could be amplified by modifying the FISH process. In this study, fluorophore-conjugated FISH probes were introduced before in situ hydrogel synthesis; hence, a significant amount of fluorescence was lost due to chemical bleaching during the subsequent free-radical polymerization process. We found that the signal intensity of mRNA puncta after a 3.7-fold expansion was almost 10 times lower than it was before the expansion due to chemical bleaching. To achieve higher FISH signals after expansion, mRNA molecules or signal amplification moiety-bearing FISH probes could be anchored directly to a hydrogel network, and subsequently, fluorophore bearing imagers or amplifiers could be introduced after hydrogel synthesis. Second, FISH-IF could be combined with a higher-resolution ExM technique, such as iterative expansion microscopy (iExM)^26^. Once combined, it can measure the mRNA expression levels more precisely and visualize the colocalization of mRNAs and proteins more clearly. As FISH-IF combined with ExM, demonstrated in this study, can be combined with other types of ExM techniques without any significant change, it is possible to achieve a higher level of multiplexed protein imaging along with the visualization of mRNAs.

## Materials and methods

### Cell culture and fixation

NIH-3T3 (purchased from Korean Cell Line Bank (KCLB), Seoul, Korea) cells were cultured on CS16-culture well removable chambered coverglass (Gracebiolabs, Bend, OR, USA) in Dulbecco’s modified eagles’ medium (DMEM; Thermofisher, Waltham, MA, USA), supplemented with 10% bovine calf serum (BCS; Thermofisher), and 1 × penicillin–streptomycin (Thermofisher). Cultured cells were briefly rinsed with 1 × PBS twice, fixed with 4% paraformaldehyde (PFA) in 1 × PBS, and washed three times with 1 × PBS. Fixed cells were stored at 4 °C until use.

### Mouse perfusion

All methods related to animal care and welfare were approved by the Korea Advanced Institute of Science and Technology Institutional Animal Care and Use Committee (KAIST-IACUC) and conducted under the guidelines and regulations in accordance with the same organization. Mice used in this study were maintained in a specific pathogen-free facility of KAIST Laboratory Animal Resource Center. All methods related to the animal studies were carried out in compliance with the ARRIVE (Animal Research: Reporting of In Vivo Experiments) guidelines. C57BL/6J mice, 5–10 weeks of age, were used for this research. Mice were anesthetized with isoflurane and then transcardially perfused with ice-cold 4% PFA in 1 × PBS. Brains were extracted from the skull and stored in 4% PFA in 1 × PBS at 4 °C for further fixation for 2 h. Fixed brains were then sliced to a thickness of 100–300 µm with a vibratome (Leica VT1000S, Wetzlar, Germany). Slices were stored in 1 × PBS containing 0.1 M glycine before use. All reagents used during experimental procedures including perfusion were made RNase-free and controlled under RNase-free condition.

### Preparation of AcX and LabelX

Acryloyl-X, SE (6-((acryloyl)amino)hexanoic acid, succinimidyl ester, hereafter abbreviated to AcX; Thermofisher) was resuspended in anhydrous dimethyl sulfoxide (DMSO) at a concentration of 10 mg/mL. Resuspended AcX was aliquoted and stored frozen at − 20 °C until use. Label-IT Amine Modifying Reagent (Mirus Bio, Madison, WI, USA) was resuspended in the manufacturer-provided reconstitution solution at a concentration of 1 mg/mL. Resuspended Label-IT Amine was reacted with AcX at a ratio of 10:1 overnight with shaking at room temperature overnight to make LabelX. For example, to react (100 µL) of resuspended Label-IT Amine, (10 µL) of resuspended AcX was reacted. Reacted LabelX was then aliquoted and stored frozen at − 20 °C in a dry environment.

### RNA-FISH for cultured cells and brain slices

All experimental procedures were performed at room tempertuare (RT), unless otherwise indicated. RNA-FISH was performed in fixed cultured cells with RNAscope (ACD [Advanced Cell Diagnostics], Bio-techne, Minneapolis, MN, USA), which was used as instructed by the manufacturer’s user manual. The fixed brain slices were dehydrated with ice-cold 50%, 70%, and 100% ethanol for 1 h each, followed by final dehydration with fresh ice-cold 100% ethanol for 1 h. Dehydrated slices were then treated with pre-heated RNAscope-protease-IV (ACD) for 1 h at RT, followed by 15 min of 1 × PBS wash twice. The RNAscope probe (ACD) was then hybridized for 2 h at 40 °C in a hybEZ oven (ACD) and washed twice for 15 min with 1 × RNAscope wash buffer (ACD). RNAscope amplification buffer 1 (ACD) was then treated for 30 min to 1 h at 40 °C in a hybEZ oven, followed by 15 min of washing twice with 1 × RNAscope wash buffer. RNAscope amplification buffer 2 (ACD) was treated for 15 min to 30 min at 40 °C in the hybEZ oven, followed by 15 min of washing twice with 1 × RNAscope wash buffer. After the second amplification, RNAscope amplification buffer 3 (ACD) was treated for 30 min to 1 h at 40 °C in the hybEZ oven, followed by 15 min of washing twice with 1 × RNAscope wash buffer. Finally, RNAscope amplification buffer 4 (ACD) was treated at 40 °C in the hybEZ oven, followed by 15 min of washing twice with 1 × RNAscope wash buffer. RNAscope DAPI (ACD) was then stained for 1 h for brain tissue slices.

### Analysis of RNA-FISH signals

RNAscope images were taken with a spinning disk confocal microscope, and then maximum-intensity Z-projection images were created with FIJI-imageJ. ‘Spotcount’ plugin FIJI-imageJ was used for counting RNA puncta. RNA puncta were also counted and localized with ‘Starsearch,’ a code developed by the Raj lab and available online. Image analysis using ‘Spotcount’ was performed in Fig. 1, and ‘Starsearch’ was used for image analysis in Fig. 3. Individual RNA-FISH puncta from before the expansion were first counted in the projection images acquired from the z-stack images of cultured cells. After the expansion, the z-stack images of the same cells, now expanded, were acquired, and the RNA-FISH puncta were counted in the projection images. Each post-exapnsion RNA punctum was then marked as ‘retained’ when the punctum, or expansion-induced resolved puncta, were observed post-expansion at the same location. Data plots were created with the Origin software (OriginLab corporation, Northampton, MA, USA) for Figs. 1d, 2c, 3g, and 5s.

### Antibody staining of proteins and post-fixation

For cultured cells, either before or after RNAscope, cells were incubated with NGS blocking buffer (5% normal goat serum [NGS], 0.1% Triton X-100, and 1 × PBS) at room temperature for 30 min for blocking and permeabilization. Then, cells were incubated with primary antibodies at a concentration of 10 µg/mL in NGS blocking buffer at room temperature for 40 min and then washed three times for 5 min with NGS blocking buffer. After primary antibody staining, cells were incubated with secondary antibodies at a concentration of 10 µg/mL in NGS blocking buffer at room temperature for 40 min and then washed three times for 5 min with NGS blocking buffer. For brain slices, either before or after RNAscope, tissues were incubated with NGS blocking buffer at room temperature for 1 h for blocking and permeabilization. Then, brain slices were incubated with primary antibodies at a concentration of 10 µg/mL in NGS blocking buffer at room temperature for 90 min and then washed three times for 30 min with NGS blocking buffer. After primary antibody staining, brain slices were incubated with secondary antibodies at a concentration of 50 µg/mL in NGS blocking buffer at room temperature for 90 min and then washed three times for 30 min with NGS blocking buffer. The secondary antibodies used were goat-anti-chicken CF633 (Biotium), goat-anti-rabbit Alexa 546 (Life Technologies, Carlsbad, CA, USA), and goat-anti-rabbit CF 405S (Biotium). When immunostaining was performed before RNA-FISH, specimens were fixed before dehydration for RNA-FISH, with 4% PFA in 1 × PBS at room temperature for 10 min for cells and 1 h for brain slices followed by washing with 0.1 M glycine in 1 × PBS for 5 min for cells and 30 min for brain slices.

### LabelX and AcX treatment of cultured cells and brain slices

LabelX was treated either after RNAscope or after immunostaining, which was performed after RNAscope. Specimens were pre-incubated in MOPS buffer (20 mm MOPS, pH 7.7) at room temperature for 30 min. Then, pre-incubated specimens were incubated with diluted LabelX at the desired concentration at 37 °C for at least 2 h for cultured cells and 6 h for brain slices. LabelX was diluted to a final concentration of Label-IT Amine concentration of 0.006 mg/mL for cells and 0.045 mg/mL for brain slices. LabelX-incubated specimens were washed twice with 1 × PBS for 5 min for cells and 30 min for brain slices. Then, specimens were incubated with AcX diluted to a final concentration of 0.1 mg/mL in 1 × PBS for at least 2 h for cultured cells and 6 h for brain slices. AcX-incubated specimens were washed twice with 1 × PBS for 5 min for cells and 30 min for brain slices.

### Gelation, digestion, and expansion

A gelation cocktail consisting of monomers, crosslinker, and salts (8.625% [w/w] sodium acrylate, 2.5% [w/w] acrylamide, 0.15% [w/w] *N,N*′-methylenebisacrylamide, 2 M NaCl, 1 × PBS) was mixed, aliquoted, and stored frozen at − 20 °C before use. For the gelation of specimens after anchoring chemical (LabelX, AcX) treatment, ammonium persulfate (APS) and tetramethylethylenediamine (TEMED) were added from 10% (w/w) and 10% (v/v) stock solutions, respectively, to achieve final concentrations of 0.2% (w/w) for APS and 0.2% (v/v) for TEMED in the gelation cocktail. Additionally, 4-hydroxy-TEMPO (H-TEMPO) was added at a final concentration of 0.01% (w/w). Specimens were then incubated in the final gelation cocktail at 4 °C for 30 min twice followed by the gelation of the specimen described in the original ExM protocol. The hydrogel-specimen composite was then homogenized in digestion buffer (1 M NaCl, 1 mM EDTA, 0.5% Triton X-100, 50 mM Tris) with proteinase K (New Engand Biolabs, Ipswich, MA, USA) diluted to a final concentration of 8 U/mL. The homogenized composite was then expanded in 1 × PBS to produce a 2.1-fold expansion, 0.1 × PBS to produce a 3.7-fold expansion, and DI water to produce a 4.5-fold expansion.

### HCR buffer treatment of cultured cells

Fixed cells are permeabilized with 70% ethanol for 12–16 h at − 20 °C and stored in freshly prepared 70% ethanol − 20 °C until use; storage of cells did not exceed a week. Stored cells were washed with 2 × SSC twice. After washing, cells were hybridized in HCR probe hybridization buffer (Molecular Instruments, Sunnyvale, CA, USA) for 12–16 h at 37 °C without any RNA-FISH probe contained. Cells were then washed with an HCR probe wash buffer 4 times, each for 5 min at 37 °C, followed by subsequent washing with 5 × SSCT (5 × SSC, 0.1% Tween 20) for 5 min twice. Washed cells were then treated with HCR amplification buffer (Molecular Instruments) for 12–16 h at room temperature followed by 5 min washing with 5 × SSCT 5 times. Cells were then subjected to immunofluorescence. The IF image of HCR buffer-treated cells is shown in Supplementary Fig. 1.

### Imaging of specimens

Both cultured cells and brain slices before expansion were imaged on a Nikon Ti-2 microscope (Tokyo, Japan) with an Andor Dragonfly 200 spinning disk confocal microscope system (Andor, Oxford instruments, Belfast, UK), controlled by the Fusion software. Expanded gels were first attached on a coverglass with a size of 48 × 60 mm coated with poly-l-lysine, followed by image acquisition. Post-expansion confocal and widefield imaging was performed on an Andor Dragonfly200 spinning disk confocal microscope system with a 40 × 1.15 NA water immersion objective lens on a Nikon Ti-2 microscope body with a 4.2 Zyla sCMOS camera (Andor). The expansion factor was calculated by imaging the same region of the specimen pre- and post-expansion. Then, the length of the same landmark structures from the image, obtained pre- and post-expansion, was compared and calculated using FIJI-imageJ.

### Supplementary Information

Supplementary Information.

Supplementary Video S1.
