# Author Correction: Simultaneous expansion microscopy imaging of proteins and mRNAs via dual-ExM

**DOI:** 10.1038/s41598-024-65252-5

**Published:** 2024-06-24

**Authors:** In Cho, Jae‑Byum Chang

**Affiliations:** Department of Materials Science and Engineering, Korea Advanced Institute of Science and Technology (KAIST), Daejeon, 34141 Republic of Korea

Correction to: *Scientific Reports* 10.1038/s41598-022-06,903-3, published online 01 March 2022

The original version of this Article contained an error in the Materials and Methods section, under the subheading ‘Gelation, digestion and expansion’.

In the original version of this Article, the gelation procedure of specimens after anchoring chemical (LabelX, AcX) treatment contained an incorrect concentration.

“For the gelation of specimens after anchoring chemical (LabelX, AcX) treatment, ammonium persulfate (APS), tetramethylethylenediamine (TEMED), and 4-hydroxy-TEMPO (H-TEMPO) were added to the gelation cocktail to final concentrations of 10% (w/w), 10% (v/w), and 0.01% (w/w), respectively.”

now reads,

“For the gelation of specimens after anchoring chemical (LabelX, AcX) treatment, ammonium persulfate (APS) and tetramethylethylenediamine (TEMED) were added from 10% (w/w) and 10% (v/v) stock solutions, respectively, to achieve final concentrations of 0.2% (w/w) for APS and 0.2% (v/v) for TEMED in the gelation cocktail. Additionally, 4-hydroxy-TEMPO (H-TEMPO) was added at a final concentration of 0.01% (w/w).”

The original Article has been corrected.

