# Supplementary material for: Simultaneous expansion microscopy imaging of proteins and mRNAs via dual-ExM
Source: Sci Rep. 2022 Mar 1;12:3360. doi: 10.1038/s41598-022-06903-3 (PMC8888644; doi:10.1038/s41598-022-06903-3)
Supplement: Supplementary file 1 — Supplementary Information. [file 41598_2022_6903_MOESM1_ESM.docx]

**Simultaneous expansion microscopy imaging of proteins and mRNAs *via* dual-ExM**

In Cho^1^, and Jae-Byum Chang^1^*

^1^Department of Materials Science and Engineering, Korea Advanced Institute of Science and Technology, Daejeon, Korea

*indicates the corresponding author


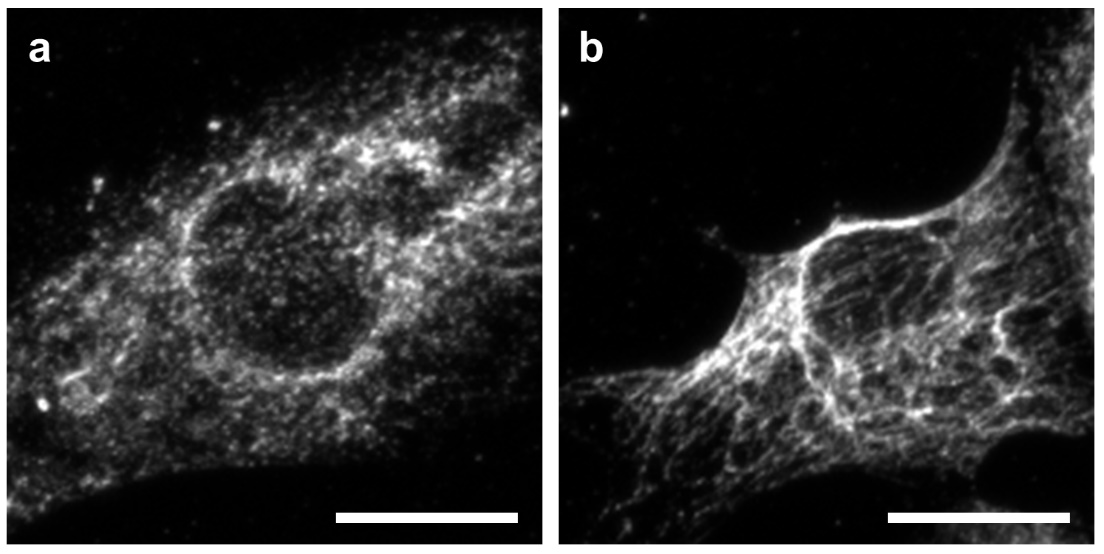
Supplementary Figure 1. Confocal microscopy image of NIH-3T3 cells labeled with an antibody against vimentin after the treatment of different FISH buffers. (a) After the treatment of a HCR buffer. (b) After the treatment of a RNAscope buffer. Scale bars in (a–b): 20 μm.


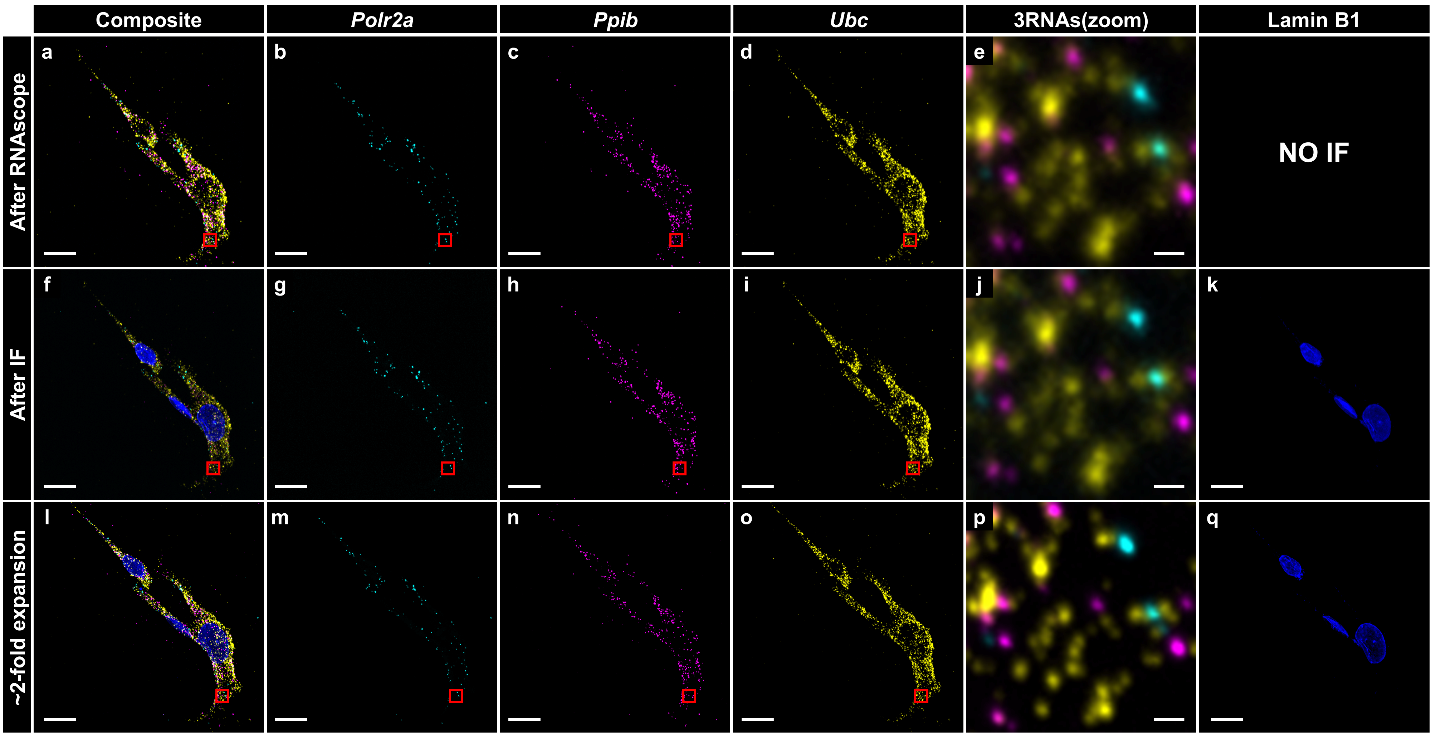


**Supplementary Figure 2. Dual-ExM with multiplexed RNA targets.** MIP of z-stack images of NIH-3T3 cells labeled with RNAscope probes against *Polr2a* mRNA (cyan), *Ppib* mRNA (magenta), *Ubc* mRNA (yellow), and antibody against Lamin B1 (blue). **a**–**e**, after RNAscope; **f**–**k**, after immunostaining of Lamin B1; **l**–**q**, after 2.1-fold expansion in 1× PBS. **a**, **f**, **l**, composite images of 3 mRNAs and Lamin B1**;** **b**, **g**, **m**, images showing only *Polr2a* mRNA; **c**, **h**, **n**, images showing only *Ppib* mRNA; **d**, **i**, **o**, images showing only *Ubc* mRNA; **e**, **j**, **p**, magnified view of the red boxed regions of each row; **k**, **q**, images showing only Lamin B1. Scale bars: (**a**–**d**), (**f**–**i**), (**k**–**o**), **q**: 20 μm; (**e**, **j**, **p**) 1 μm. All length scales are presented in pre-expansion dimensions.

**
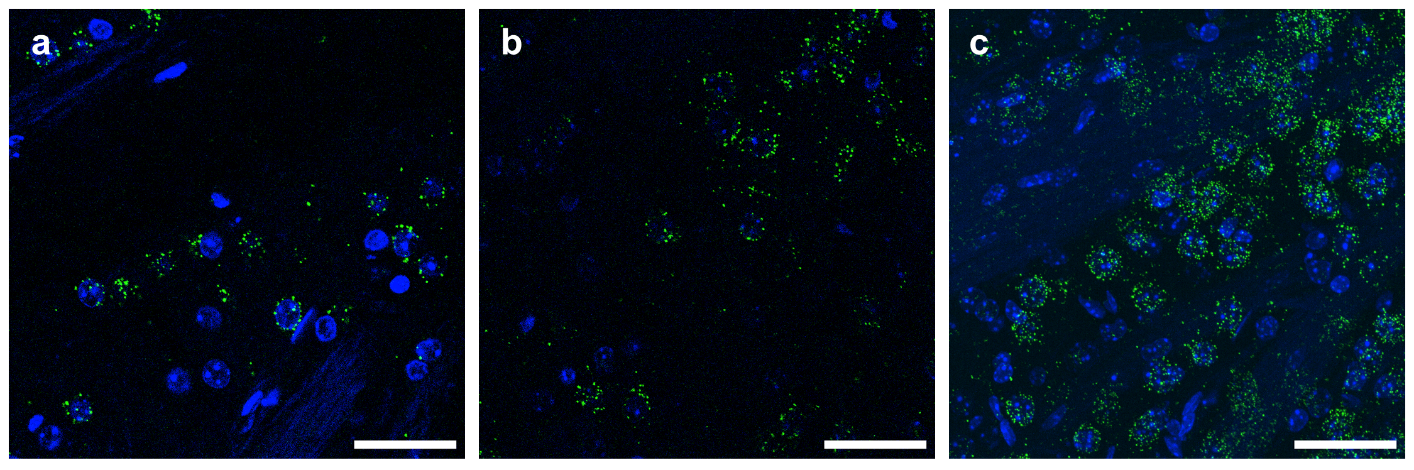
Supplementary Figure 3. RNAscope labeling of a thick mouse brain slice.** (**a**) Confocal microscopy image of DAPI (blue) and *DRD1* mRNA (green) in the striatum at the surface of a mouse brain slice. (**b**) Same brain slice in **a**, but image acquired at 50 μm below the surface. (**c**) MIP of a z-stack image of the mouse brain shown in **a**, **b**. The z-stack image was acquired from the surface to 50 μm deep in the tissue. Scale bars in (**a**–**c**): 40 μm.

**
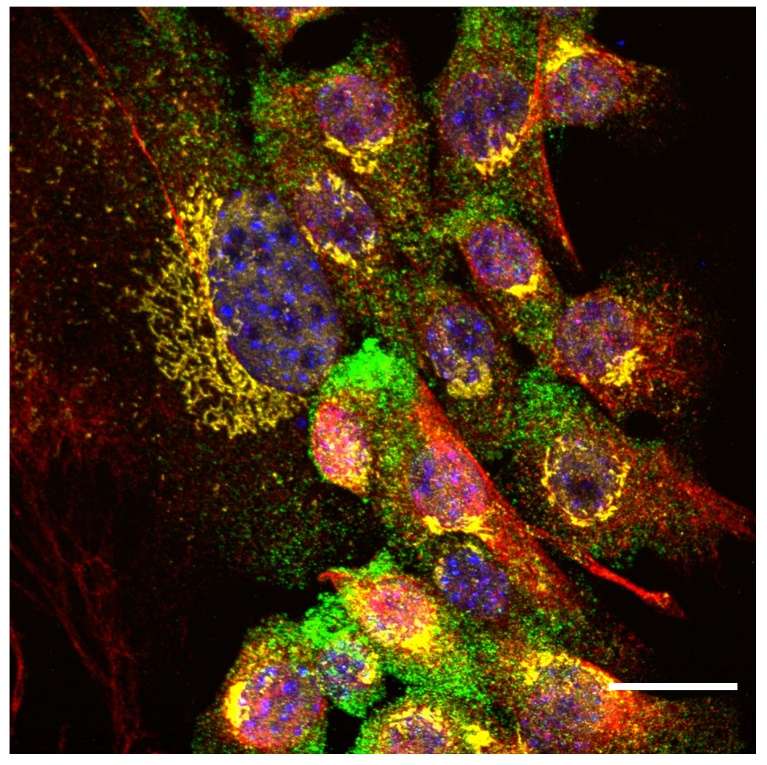
Supplementary Figure 4. Dual ExM imaging of cultured cells, prepared with the IF-FISH process.** MIP of a z-stack image of 2.1-fold expanded cultured NIH-3T3 cells, labeled with antibodies against vimentin (red) and GM130 (yellow), a RNAscope probe against *Gapdh* mRNA (green), and DAPI (blue). The IF-FISH process was used to label both proteins and mRNAs. Scale bar: 25 μm. Scale is presented in pre-expansion dimension.

**Supplementary
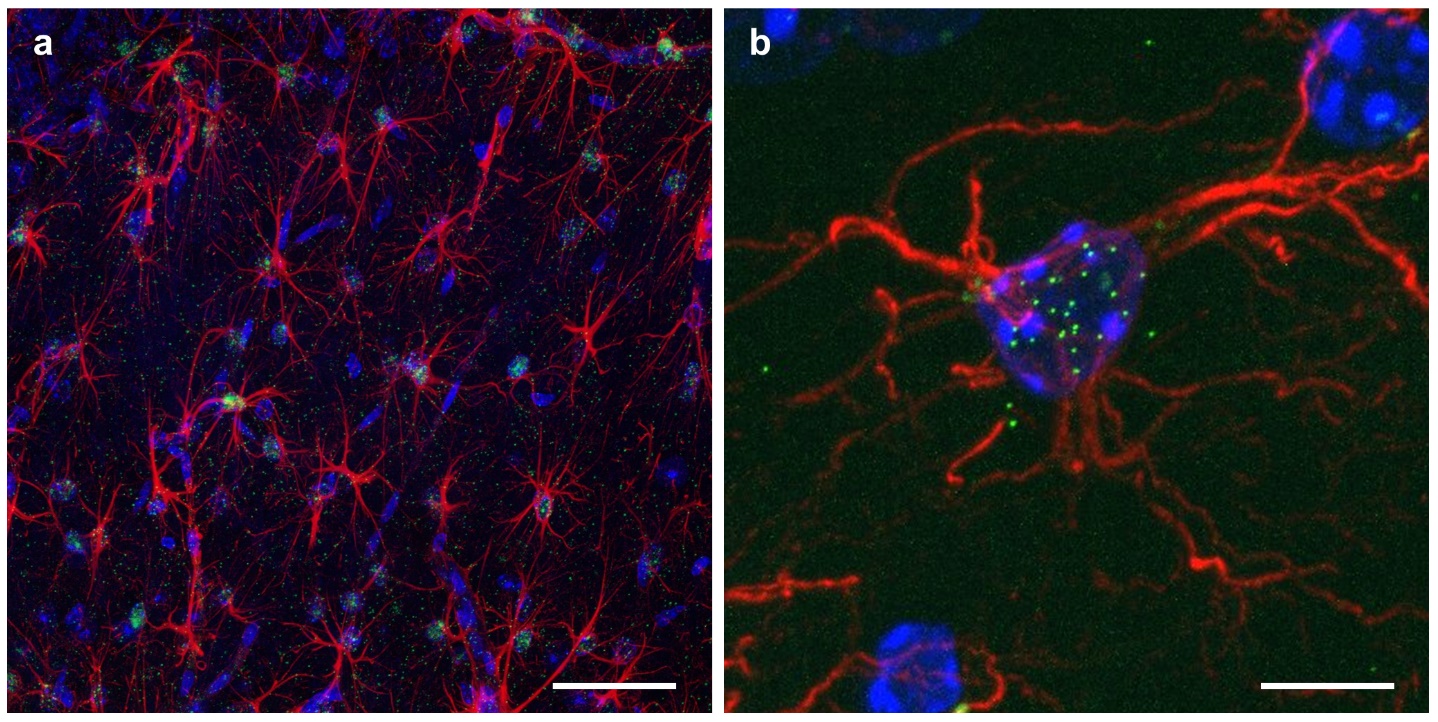
Figure 5. Dual ExM imaging of a mouse brain slice prepared with the IF-FISH process.** MIP of z-stack images of a mouse brain slice, labeled with a RNAscope probe against *Gfap* mRNA (green), an antibody against GFAP (red), and DAPI (blue). The IF-FISH process was used to label both proteins and mRNAs. (**a**) Before expansion. (**b**) After 2.1‑fold expansion. Scale bars: (**a**) 50 μm; (**b**) 10 μm. All length scales are presented in pre-expansion dimensions.

**Supplementary Video 1. 3D dual-ExM imaging of a mouse brain slice.** 3D reconstructed images of z-stacks of a mouse brain slice, labeled with a RNAscope probe against *Gfap* mRNA (green), an antibody against GFAP (red), and DAPI (blue). Left: before expansion; right: after 2.1‑fold expansion. Grid unit: 10 μm.
